# Immune cell changes following chemotherapy in advanced pancreatic cancer with variations based on gender

**DOI:** 10.1038/s41598-025-26219-2

**Published:** 2025-11-22

**Authors:** Roberto Aquilani, Salvatore Corallo, Roberto Maestri, Silvia Brugnatelli, Paolo Iadarola, Simona Viglio, Simone Figini, Beatrice Filippi, Domiziana Alaimo, Silvio Sporeni, Manuela Verri, Federica Boschi

**Affiliations:** 1 Department of Biology and Biotechnology “Lazzaro Spallanzani”, University of Pavia, Via Ferrata 9, 27100 Pavia, Italy; 2 Department of Internal Medicine and Medical Therapy, Via Ferrata 1, 27100 Pavia, Italy; 3 SC Oncology Unit, IRCCS Foundation, San Matteo Hospital, Largo C. Golgi 19, 27100 Pavia, Italy; 4 Department of Biomedical Engineering, Istituti Clinici Scientifici Maugeri IRCCS, Scientific Institute of Montescano, 27040 Montescano, Italy; 5 Department of Molecular Medicine, Biochemistry Unit, University of Pavia, Viale T. Taramelli 3, 27100 Pavia, Italy; 6 Department of Drug Sciences, University of Pavia, Viale T. Taramelli, 14, 27100 Pavia, Italy

**Keywords:** Pancreatic cancer, Chemotherapy changes in circulating immune cells, Gender, Predictors of survival, Cancer, Oncology

## Abstract

To explore the changes in blood inflammatory cells (neutrophils, monocytes, platelets) and adaptive immune cells (lymphocytes) during chemotherapy, we retrospectively analysed medical records from 66 patients with unresectable Pancreatic Ductal Adenocarcinoma (PDAC) treated with the Gemcitabine-nab-Paclitaxel (GnP) regimen. Evaluations were conducted at baseline (pre-GnP, TA), after the first cycle (TB), and after the third cycle (TC) of treatment. In metastatic PDAC (mPDAC), the monocyte-to-lymphocyte ratio significantly increased at both TB and TC compared to TA, whereas no such change was observed in locally advanced PDAC (laPDAC) (interaction: p = 0.006). Platelet levels rose over time in both phenotypes, with a more pronounced increase in mPDAC (intergroup: p = 0.008). When stratified by gender, males with mPDAC showed an increase in monocyte percentages among total white blood cells (intergroup: p = 0.018), while both phenotypes exhibited rising platelet-to-lymphocyte ratios over time. In females, the platelet-to-lymphocyte ratio increased more significantly in laPDAC than in mPDAC (interaction: p = 0.046). GnP treatment notably increased circulating inflammatory cells and their relationships with lymphocytes in a manner dependent on both disease phenotype and gender. Pretreatment factors such as monocyte counts < 0.6 × 10^3^/µl, lymphocyte counts > 1 × 10^3^/µl, and a monocyte-to-lymphocyte ratio < 0.43 were identified as independent predictors of survival.

## Introduction

The benefits of anticancer treatments (surgery, chemotherapy, radiotherapy) in cancer patients may be counterbalanced by inflammation within the tumour microenvironment (TME) caused by tumour cells killed during treatment. If not adequately cleared by phagocytic activity, dead cells release pro-inflammatory and pro-angiogenic cytokines and eicosanoids^1–7^, promoting the proliferation of surviving tumour cells, increased cancer cell numbers^8^, tumor growth, invasion, and metastasis^8–10^. High levels of tumour cell death are associated with metastasis and poor outcomes^11–16^.

In mouse models treated with Gemcitabine, cancer cell death stimulated primary hepatopancreatic cancer growth and metastasis when co-injected with an amount of non-tumorigenic cancer cells. This process was mediated by activation of soluble Eicosanoid Hydroxylase (sEH) and Prostaglandin E2 receptor 4 (PE4) pathways^17^. Pharmacologic inhibition of sEH and PE4 prevented cancer progression by enhancing tumour cell clearance through phagocytosis and reducing pro-inflammatory cytokine and eicosanoid production in the TME^17^.

Macrophages, a key phagocytic cell population in the TME, are heterogeneous and plastic cells^18^. From among the macrophages with different origin^19^, those derived from circulating monocytes form the most prominent subset^20^. Tumours can produce colony-stimulating factors that promote the production and recruitment of myeloid cells including monocytes, into the TME^19^. However, recruited monocytes and monocyte-derived macrophages undergo functional changes^19,21^ often become immunosuppressive due to the production of tumour-derived IL-10, TGFβ, and PGE, thus contributing to immune evasion^20,22–26^. In plasma of pancreatic ductal adenocarcinoma (PDAC) patients, circulating monocyte levels correlate with increased concentrations of TNFα and IL-6, indicating disease severity^27^. Experimental and clinical studies emphasize the role of monocytes in cancer progression. For example, murine breast cancer models show improved outcomes after depleting circulating monocytes^28^. Clinically, circulating monocytes are prognostic in PDAC^27^, although in murine pancreatic cancer models TME-resident macrophages, rather than monocyte-derived macrophages, primarily support tumour growth in pancreatic cancer models^29^. In preclinical investigations, macrophages negatively reduced the responsiveness to chemotherapy^19^.

Neutrophils, another TME-recruited cell type, exhibit dual roles^30,31^. While they can promote tumour growth, angiogenesis, and metastasis^30–36^, they also exert toxic effects on tumours through the production of nitric oxide (NO) and reactive oxygen species (ROS). High neutrophil levels and neutrophil-to-lymphocyte ratios (NLR) are generally associated with poor prognosis and chemotherapy resistance in pancreatic cancer^31,37^. Gemcitabine may exacerbate inflammation by inducing IL-1β secretion in neutrophils^38^ but also enhances adaptive immune responses by inducing neutrophil apoptosis (as does 5-FU and Doxorubicin)^39,40^.

Developing drugs to reprogram monocyte/macrophage functions and enhance dead-cell clearance in the TME could reduce cancer recurrence^17,41^. Until these drugs become widely available, understanding the impact of chemotherapy on systemic blood cell counts and their relationships could help evaluate the inflammatory and immune responses induced by a given chemotherapy treatment.

This study investigated the effects of Gemcitabine-nab-Paclitaxel (GnP) on blood cell counts in patients with advanced PDAC, hypothesizing that metastatic PDAC (mPDAC) patients would exhibit greater increases in inflammatory monocytes than those with locally advanced PDAC (laPDAC). This hypothesis was based on the ability of nab-Paclitaxel to enhance intra-tumoral Gemcitabine (G) concentrations^42–44^, increasing tumour cell death and necessitating higher macrophage clearance activity. Consequently, elevated blood monocyte levels could promote metastasis^27,45–51^.

The study also analysed GnP’s impact on systemic inflammation markers, including NLR, monocyte-to-lymphocyte ratio (MLR), and platelet-to-lymphocyte ratio (PLTLR), as well as their associations with gender. Finally, the potential of blood cell counts as survival predictors in PDAC patients was assessed^52–55^.

## Materials and methods

### Patients

This observational retrospective study was approved by the Ethical Committee of Policlinico S. Matteo (Pavia, Italy; P-20210006621, 4 June 2021). All procedures were performed in compliance with relevant guidelines and regulations and have been approved by the appropriate institutional committee(s). Sixty-six patients with a stage IV PDAC diagnosis, admitted to our oncology department from January 2021 to September 2022 were considered in this retrospective observational study. Patients’ medical records were retrieved from the database of the Department. The inclusion criteria were: (1) histological/cytological diagnosis of non-resettable exocrine pancreatic cancer (any T, any NM1 and T4); (2) age > 18 years; (3) Karnofsky Index > 70; (4) haemoglobin concentration > 9 g/dl; (5) serum creatinine < 1,5 mg/dl; (6) serum albumin > 3,0 g/dl; (7) patients on first-line chemotherapy following the AIOM 2020 Guidelines. Only patients on GnP as first-line treatment of PDAC were included. GnP regime consisted in the administration of nab-Paclitaxel (125 mg per square meter of body surface area) followed by Gemcitabine 100 mg *per* square meter on day 1 day 8 and day 15 every 4 weeks for 7–8 weeks. Patients received GnP until disease progression. The exclusion criteria were the following: (1) patients with potentially resettable cancer; (2) patients with islet cells cancer; (3) patients < 18 years; (4) patients without any previous chemotherapy; (5) patients with cardiovascular disease and hemodynamic instability; (6) presence of collagenopathies; (7) patients with diabetes mellitus with poor metabolic control; (8) patients who had not completed 3 cycles of chemotherapy.

### Materials and procedures

Demographic-, anthropometric-, laboratory data, oncologic data, including Computed Tomography examination (CT) were considered. Patients were subsequently categorized in phenotypes mPDAC (n = 53, 80,3%) and laPDAC (n = 13, 19,7%). Patients were monitored until death or admission to a palliative care setting. The elapsed time (months, mo) until death was calculated from the beginning of the first cycle of chemotherapy.

### Procedures

The patients’ peripheral White Blood Cell (WBC) counts and cell subpopulations were determined in pre-treatment period (Time A[TA]; within 3 days before Cycle 1, day 1), after the end of first cycle of GnP therapy (Time B[TB]: 28 ± 7 days after Cycle 1, day 1) and after three months (3 cycles)of GnP therapy (Time C[TC]: 84 ± 7 days after Cycle 1, day 1).

At TA, the profiles of WBCs and cell subpopulations were compared with those of 18 healthy subjects (Controls: Contr.) recruited as having similar age, body mass index (BMI, kg/m^2^) and not suffering from any disease, with sedentary lifestyle. Blood samples for determination of WBCs and other bio humoral variables had been drown at 8:a.m. after 12-h overnight fasting.

From the WBC subpopulations cells, we calculated the following ratios:

1. Neutrophil (N) to Lymphocyte (L) ratio (NLR), an indicator of systemic inflammation and established prognostic factor in patients with cancer^56,57^.
2. Monocyte (M) to Lymphocyte ratio (MLR), an indicator of inflammation in several disease including colorectal cancer^58^, pancreatic cancer^59,60^. NLR and MLR is the current investigation served as factors describing a general balance between innate immune response to adaptive immune response. The prevalence of inflammatory cells (N, M) over L, impairment of adaptive immune response influences the progression of the cancer, as they are responsible for reducing immunosurveillance and dissemination of circulating cancer cells^61^.
3. Platelet (PLT) to Lymphocyte ratio (PLTLR). PLT influence cancer progression, mainly tumour cell metastasis^62–65^.

These measurements were done on the entire PDAC population, on mPDAC and laPDAC subpopulations and on mPDAC and laPDAC stratified for gender (males/females). since sex hormones are known to influence the immune system. Additionally, the WBCs counts of subpopulations and subpopulation ratios were done on patients who died before six months after diagnosis (< 6 mo), between 6 to 12 months after diagnosis (6–12 mo) and beyond 12 months after diagnosis (> 12 mo).

### Objective

The primary focus of this work was to highlight the increase of counts (in absolute amount or in percentage of WBC counts) of circulating monocyte during cycles of GnP mainly in mPDAC compared to laPDAC subpopulations, also after stratification for gender.

### Statistical analysis

Descriptive statistics are presented as mean ± SD for continuous variables and as number (n) and percentage (%) for discrete variables. The time course (TA, TB, TC) of the considered variables was investigated by repeated measures analysis of variance (ANOVA). Post hoc analysis (Tukey–Kramer criterion) was performed for significant results from ANOVA. The chi-squared test or Fisher’s exact test, as appropriate, was used to compare dichotomous variables. The comparison of the time course of the studied variables between the mPDAC and laPDAC subgroups was investigated using a two-factor analysis of variance, where the first factor (between factor) was group (mPDAC and laPDAC) and the second factor (within factor) was time (three measurements, TA, TB, TC), with repeated measurements in the time factor. The focus of this analysis was on the interaction term (time × group). An overall significant interaction effect was followed up by post-hoc analysis (Tukey’s HSD test). The same analysis was performed for all patients and after stratification by sex. Comparison of variables at the three time points between patients who died within 6 months of diagnosis, those who died between 6 and 12 months of diagnosis, and those who survived more than 12 months of diagnosis was performed by ANOVA, followed by post-hoc analysis in the case of significant differences.

To assess the association between selected variables and survival outcomes, the variables were dichotomized using the median value as the cutoff. We chose the median cut-off because we were concerned that, given the relatively small sample size, data-driven optimization of the cut-off could substantially inflate the risk of overfitting and type I error and would likely result in unstable thresholds that are difficult to reproduce in independent cohorts.

Accordingly, patients were divided into two groups: those with values less than or equal to the median and those with values above the median. One-year survival curves for the two groups were estimated using the Kaplan–Meier method and compared using the log-rank test.

The relationship between continuous variables was assessed by Spearman’s rank correlation. All tests were two-tailed. A p-value < 0.05 was considered statistically significant. All statistical analyses were performed with the SAS/STAT statistical package, version 9.4 (SAS Institute Inc., Cary, NC, USA).

## Results

Sixty-six Caucasian patients who received GnP as first-line treatment for unresectable, locally advanced or metastatic PADC were included in the analysis. The median age of the patients was 62.4 years, and 38 were men (57.5%). At the beginning of treatment, 19.7% (n = 13) were diagnosed with locally advanced stage disease, while 80.3% (n = 53) had metastatic stage disease. Patients and disease characteristics are shown in Table 1.

**Table 1:** Clinical characteristics of the study patients with pancreatic ductal adenocarcinoma cancer (n = 66).

Demography	
Age (years)	62.4 ± 12.4
Males/females (%)	57.5/42.5
Anthropometry	
Body Mass Index (kg/m^2^)	%
22.6 ± 4.1 of whom	
< 18.5	12.2
18.5–24.99	65.1
25.0–29.99	15.1
≥ 30	7.6
ECOG* performance status N (%)	
0	36 (54.5)
1	30 (45.5)
Stage N (%)	
Locally advanced	13 (19.7)
Metastatic	53 (80.3)
Metastasis site (%)	
Liver	60.4
Peritoneum	17
Lung	11.3
Other	11.3
Grade ≥ 1 treatment-related toxicities (%)	
Neurological	13.56
Ematological	15.1
Asthenia	10.6
Diarrhea	3
Mucositis	1.5
Gemcitabine and/or NabPaclitaxel dose reductions (%)	40.9

*ECOG = Eastern Cooperative Oncology Groups Performance Status.

Table 2 shows the bio humoral variables found at TA, at TB and at TC. Table 3 describes statistical comparisons between patients and controls in circulating N, M, L, PLT and NLR, MLR, PLTR.

**Table 2:** Blood immune cells variables found at pre-treatment (TA), after 1 month of treatment (TB) and 3 months treatment (TC) time points.

Variables (nv)	TA	TB	TC	p time	TA vs TB p value	TA vs TC p value	TB vs TC p value
Haemoglobin g/dl (nv 13.2–17.3)	12.27 ± 1.67	11.17 ± 1.31	11.00 ± 1.46	< 0.0001	< 0.0001	< 0.0001	0.61
Red blood cells 10^6^/µl (nv 4.30–5.70)	4.15 ± 0.63	3.73 ± 0.48	3.66 ± 0.52	< 0.0001	< 0.0001	< 0.0001	0.41
Haematocrit % (nv 39.0–49.0)	36.4 ± 5.0	33.2 ± 4.1	33.0 ± 3.8	< 0.0001	< 0.0001	< 0.0001	0.95
WBC 10^3^/µl (nv 4.00–10.00)	7.69 ± 3.16	7.46 ± 3.80	8.40 ± 5.28	0.30			
Neutrophils 10^3^/µl (nv 2.0–8.0)	5.428 ± 2.702	4.893 ± 2.708	6.235 ± 4.996	0.053	0.34	0.43	0.069
Neutrophils %	68.9 ± 10.1	65.9 ± 12.8	69.3 ± 15.2	0.13			
Lymphocytes 10^3^/µl (nv 1.5–4.0)	1.447 ± 0.755	1.416 ± 0.846	1.348 ± 0.752	0.46			
Lymphocytes %	20.5 ± 8.8	21.7 ± 10.5	20.2 ± 12.7	0.59			
Monocyte 10^3^/µl (nv 0.1–10.0)	0.617 ± 0.333	0.776 ± 0.579	0.784 ± 0.649	0.08			
Monocyte %	8.05 ± 3.14	10.44 ± 4.80	8.89 ± 4.92	0.003	0.001	0.44	0.09
Eosinophil 10^3^/µl (nv 0.1–0.5)	0.00016 ± 0.00018	0.00028 ± 0.001.3	0.00012 ± 0.00014	0.45			
Eosinophil %	1.53 ± 1.71	1.35 ± 2.23	1.30 ± 1.65	0.71			
Basophil 10^3^/µl (nv 0.0–0.2)	0.04 ± 0.07	0.07 ± 0.16	0.05 ± 0.10	0.38			
Basophil %	0.52 ± 0.51	0.67 ± 0.49	0.61 ± 1.03	0.46			
Platelets 10^3^/µl (nv 150–450)	253.277 ± 102.384	346.492 ± 184.844	274.169 ± 143.353	0.00015	0.0006	0.45	0.017
Ratios							
NLR	4.76 ± 3.77	5.19 ± 7.72	7.07 ± 10.25	0.025	0.87	0.12	0.005
MLR	0.51 ± 0.38	0.57 ± 0.34	0.67 ± 0.60	0.10			
PLTLR	209.7 ± 117.2	342.3 ± 412.6	262.7 ± 235.6	0.001	0.013	0.07	0.049

Statistical analysis: a repeated measures ANOVA with time as a within-subject factor, including three levels corresponding to the measurements at different time points: TA, TB, and TC. Post hoc analysis (Tukey–Kramer criterion) was performed for significant results from ANOVA The level of statistical significance was set at p < 0.05.

nv = normal value.

**Table 3:** Blood immune cells and their ratios found in the study patients. Comparison between patients and heathy subjects (controls). Into brackets (min → max).

Variables	Laboratory normal values (n° × 10^3^/µl)	Healthy subjects (n° × 10^3^/µl)	Patients at the PDAC diagnosis (n° × 10^3^/µl)
WBC count	4–10	6.210 ± 1.243 (4.480–9.100)	7.689 ± 3.134^°^ (2.200–18.430)
Neutrophils count	2–8	3.329 ± 0.976 (1.997–6.124)	5.428 ± 2.702^°°^ (1.000–16.430)
Lymphocytes count	1.5–4	2.192 ± 0.390 (1.571–2.815)	1.447 ± 0.755^°°^ (0.280–4.830)
Monocyte count	0.1–1	0.510 ± 0.125 (0.302–0.818)	0.619 ± 0.330* (0.050–1.700)
Eosinophil count	0.01–0.05	–	
Basophil count	0–0.002	–	
Platelets count	150–450	260.660 ± 70.689 (172.000–419.000)	252.257 ± 101.930 (75.000–576.000)
Ratios			
NLR	–	1.55 ± 0.48 (0.89–2.66)	4.76 ± 3.77^°°^ (1.04–18.6)
MLR	**–**	0.238 ± 0.07 (0.13–0.46)	0.512 ± 0.378^°°^ (0.036–1.96)
PLTLR	**–**	122.7 ± 44 (77.26–263.5)	207.69 ± 117.41^°°^ (65.69–679.6)

Statistical analysis: comparisons performed by unpaired t-test.

^°^p < 0.003.

^°°^p < 0.0001.

*p < 0.0336.

At TA the entire patient population presented with anaemia and normal values of WBC, N, M, PLT. L counts were at lower limits of normal laboratory ranges. Compared to controls however, patients had elevated WBC, N, M, lower L, similar PLT and higher NLR, MLR, PLTLR.

At TB and TC, GnP (Table 2) caused a progressive worsening of anaemia (p < 0.0001) and increases of M%, PLT, PLR, NLR.

After stratification of the PDAC population into mPDAC and laPDAC subgroups (Table 4), progressive increases of WBC in mPDAC but not in laPDAC (intergroup difference, p = 0.025) were observed. Opposite changes were observed in the time courses of M%, with an increase in the value in mPDAC and a decrease in the values in laPDAC (interaction p = 0.006). PLT counts increased in both groups, mainly in mPDAC (intergroup difference, p = 0.008). Lymphopenia was stable in both groups. PLTLR and NLR showed similar overtime increases in both groups while pretreatment MLR changed in opposite direction, increasing in mPDAC and decreasing in laPDAC.

**Table 4:** Blood immune cells variables found in entire patients’ population with PDAC after stratification for locally advanced PDAC (laPDAC, n°13 patients) and metastatic PDAC (mPDAC, n°53 patients) at pre-treatment (TA), after 1 month of treatment (TB) and 3 months treatment (TC) time points.

Variables	TA laPDAC	TA mPDAC	TB laPDAC	TB mPDAC	TC laPDAC	TC mPDAC	p time	laPDAC *vs* mPDAC	interact
Haemoglobin g/dl	12.57 ± 1.50	12.17 ± 1.73	11.34 ± 0.88	11.12 ± 1.43	11.09 ± 1.11	10.97 ± 1.57	< 0.0001	0.53	0.81
Red blood cells 10^6^/µl	4.20 ± 0.55	4.14 ± 0.65	3.71 ± 0.40	3.73 ± 0.51	3.61 ± 0.37	3.67 ± 0.56	< 0.0001	0.98	0.72
Haematocrit %	36.9 ± 4.2	36.2 ± 5.2	33.2 ± 3.5	33.2 ± 4.3	32.8 ± 2.6	33.1 ± 4.1	< 0.0001	0.87	0.76
WBC 10^3^/µl	6.61 ± 1.26	7.98 ± 3.46	5.29 ± 1.71	8.06 ± 4.00	6.95 ± 3.46	8.80 ± 5.65	0.30	0.025	0.65
Neutrophils 10^3^/µl	4.639 ± 1.050	5.641 ± 2.968	3.733 ± 1.554	5.205 ± 2.875	5.142 ± 3.200	6.529 ± 5.366	0.14	0.09	0.93
Neutrophils %	70.8 ± 8.4	68.4 ± 10.5	69.4 ± 14.1	64.9 ± 12.4	70.5 ± 12.6	69.0 ± 15.9	0.44	0.35	0.79
Lymphocytes 10^3^/µl	1.309 ± 0.510	1.484 ± 0.809	1.064 ± 0.469	1.511 ± 0.901	1.187 ± 0.762	1.391 ± 0.751	0.45	0.19	0.32
Lymphocytes %	19.7 ± 7.4	20.7 ± 9.2	21.1 ± 10.1	21.8 ± 10.7	19.6 ± 9.4	20.3 ± 13.5	0.69	0.75	1.00
Monocyte 10^3^/µl	0.5043 ± 0.1811	0.6488 ± 0.3589	0.3807 ± 0.2616	0.8855 ± 0.5972	0.4793 ± 0.296.2	0.8676 ± 0.695	0.63	0.001	0.20
Monocyte %	7.72 ± 2.55	8.15 ± 3.30	7.18 ± 4.70	11.33 ± 4.47	7.52 ± 4.38	9.26 ± 5.03	0.27	0.018	0.006
Eosinophil 10^3^/µl	0.00013 ± 0.00009	0.00017 ± 0.00020	0.00007 ± 0.00005	0.00034 ± 0.00147	0.00012 ± 0.00011	0.00012 ± 0.00014	0.87	0.42	0.68
Eosinophil %	1.91 ± 1.23	1.42 ± 1.81	1.37 ± 0.80	1.35 ± 2.49	1.86 ± 1.76	1.15 ± 1.61	0.68	0.32	0.60
Basophil 10^3^/µl	0.00003 ± 0.00002	0.00005 ± 0.00008	0.00004 ± 0.00003	0.00008 ± 0.00018	0.00003 ± 0.00002	0.00005 ± 0.00012	0.68	0.26	0.90
Basophil %	0.53 ± 0.26	0.52 ± 0.56	0.94 ± 0.73	0.59 ± 0.39	0.62 ± 0.62	0.61 ± 1.12	0.23	0.38	0.41
Platelets 10^3^/µl	207.143 ± 81.271	265.941 ± 104.599	270.000 ± 127.860	367.490 ± 193.379	204.929 ± 99.994	293.176 ± 148.302	0.007	0.008	0.76
Ratios									
NLR	4.40 ± 2.77	4.86 ± 4.02	5.87 ± 8.38	5.01 ± 7.61	7.99 ± 11.73	6.83 ± 9.93	0.038	0.79	0.74
MLR	0.384 ± 0.35	0.436 ± 0.44	0.36 ± 0.21	0.586 ± 0.56	0.358 ± 0.39	0.404 ± 0.92	0.85	0.53	0.12
PLTLR	193.1 ± 152.2	214.3 ± 107.0	425.2 ± 676.7	319.6 ± 310.5	299.7 ± 422.9	252.6 ± 155.0	0.0006	0.53	0.33

Statistical analysis: two-factor analysis of variance, the first factor (between factor) being group (mPDAC and laPDAC) and the second factor (within factor) being time (three measurements, TA, TB, TC), with repeated measurements in the time factor.

After stratification of mPDAC and laPDAC for gender, males mPDAC *vs* laPDAC exhibited the alterations described in the entire mPDAC population, that is, increases in M%, PLT count, PLTLR. NLR was an exception as it increased in both groups, although the changes were not statistically significant (Table 5).

**Table 5:** Blood immune cells variables found in male patients’ population with PDAC after stratification for locally advanced PDAC (laPDAC, n°8 patients) and metastatic PDAC (mPDAC, n°30 patients) at pre-treatment (TA), after 1 month of treatment (TB) and 3 months treatment (TC) time points.

Variables	TA laPDAC	TA mPDAC	TB laPDAC	TB mPDAC	TC laPDAC	TC mPDAC	p time	laPDAC or mPDAC	Interact
Haemoglobin g/dl	13.34 ± 1.16	12.45 ± 1.91	11.76 ± 0.75	11.37 ± 1.41	11.35 ± 1.02	11.01 ± 1.62	< 0.0001	0.29	0.63
Red blood cells 10^6^/µl	4.41 ± 0.45	4.18 ± 0.61	3.85 ± 0.33	3.77 ± 0.50	3.57 ± 0.37	3.62 ± 0.55	< 0.0001	0.64	0.36
Haematocrit %	39.1 ± 3.2	37.0 ± 5.4	34.8 ± 3.0	33.7 ± 4.3	33.2 ± 2.9	32.9 ± 4.2	< 0.0001	0.40	0.58
WBC 10^3^/µl	6.65 ± 1.18	8.07 ± 3.65	5.51 ± 1.92	9.08 ± 3.89	7.62 ± 4.03	9.60 ± 6.94	0.44	0.09	0.63
Neutrophils 10^3^/µl	4.703 ± 1.138	5.818 ± 3.240	4.091 ± 1.932	5.999 ± 2.859	5.776 ± 3.621	7.283 ± 6.555	0.30	0.21	0.93
Neutrophils %	71.4 ± 9.3	69.2 ± 11.0	71.1 ± 14.6	68.5 ± 12.1	72.6 ± 11.2	69.7 ± 14.8	0.89	0.52	0.99
Lymphocytes 10^3^/µl	1.345 ± 0.437	1.430 ± 0.799	0.989 ± 0.250	1.464 ± 0.848	1.206 ± 0.878	1.408 ± 0.732	0.52	0.33	0.36
Lymphocytes %	20.6 ± 7.3	20.1 ± 9.4	20.5 ± 11.0	18.9 ± 8.7	18.2 ± 9.4	19.8 ± 12.6	0.81	0.96	0.74
Monocyte 10^3^/µl	0.4538 ± 0.1818	0.6272 ± 0.2955	0.3188 ± 0.2291	0.9100 ± 0.4991	0.4913 ± 0.3052	0.9517 ± 0.7782	0.40	0.005	0.29
Monocyte %	6.87 ± 2.50	7.85 ± 3.04	6.18 ± 4.71	11.12 ± 4.83	6.71 ± 3.45	9.13 ± 4.72	0.46	0.022	0.16
Eosinophil 10^3^/µl	0.00012 ± 0.00004	0.00014 ± 0.00014	0.00008 ± 0.00004	0.00049 ± 0.00193	0.00012 ± 0.00013	0.00012 ± 0.00011	0.81	0.52	0.72
Eosinophil %	1.83 ± 0.65	1.24 ± 1.36	1.51 ± 0.81	1.40 ± 2.16	1.95 ± 2.14	1.21 ± 1.40	0.95	0.32	0.70
Basophil 10^3^/µl	0.00003 ± 0.00002	0.00004 ± 0.00002	0.00004 ± 0.00002	0.00010 ± 0.00023	0.00003 ± 0.00002	0.00004 ± 0.00004	0.51	0.35	0.64
Basophil %	0.47 ± 0.31	0.48 ± 0.38	0.80 ± 0.51	0.54 ± 0.41	0.59 ± 0.63	0.48 ± 0.47	0.22	0.30	0.50
Platelets 10^3^/µl	187.750 ± 77.278	243.724 ± 89.353	247.875 ± 94.383	380.207 ± 167.257	2005.00 ± 106.213	282.621 ± 124.209	0.003	0.020	0.40
Ratios									
NLR	3.90 ± 1.62	5.45 ± 4.70	4.47 ± 2.22	6.37 ± 9.72	6.99 ± 7.20	7.68 ± 12.57	0.27	0.64	0.93
MLR	0.37 ± 0.19	0.53 ± 0.39	0.31 ± 0.23	0.66 ± 0.38	0.54 ± 0.50	0.82 ± 0.77	0.21	0.053	0.79
PLTLR	144.4 ± 56.5	214.2 ± 112.4	263.9 ± 121.2	371.3 ± 391.7	218.9 ± 122.8	253.0 ± 178.6	0.038	0.33	0.79

Statistical analysis: two-factor analysis of variance, the first factor (between factor) being group (mPDAC and laPDAC) and the second factor (within factor) being time (three measurements, TA, TB, TC), with repeated measurements in the time factor.

At the end of the 3^rd^ GnP cycle (TC), mPDAC patients, compared to laPDAC ones, significantly increased baseline monocyte counts (from TA 0.6297 ± 0.2955 × 10^3^/µl to TC 0.9517 ± 0.7782 × 10^3^/µl *vs* laPDAC (TA 0.4538 ± 0.1818 × 10^3^/µl to TC 0.4913 ± 0.3052 × 10^3^/µl, p = 0.038).

In females, (Table 6), only the time courses of PLTLR ratio resulted significantly different between mPDAC and laPDAC. The increase was more evident in laPDAC than in mPDAC (interaction, p = 0.046). In comparison to their respective TA values, at the end of 3^rd^ cycle of GnP, both mPDAC and laPDCA showed no differences of the variables.

**Table 6:** Blood immune cells variables found in female patients’ population with PDAC after stratification for locally advanced PDAC (laPDAC, n°6 patients) and metastatic PDAC (mPDAC, n°22 patients) at pre-treatment (TA), after 1 month of treatment (TB) and 3 months treatment (TC) time points.

Variables	TA laPDAC	TA mPDAC	TB laPDAC	TB mPDAC	TC laPDAC	TC mPDAC	p time	laPDAC or mPDAC	Interact
Haemoglobin g/dl	11.55 ± 1.32	11.72 ± 1.32	10.77 ± 0.76	10.72 ± 1.41	10.73 ± 1.21	10.91 ± 1.53	0.011	0.86	0.92
Red blood cells 10^6^/µl	3.93 ± 0.60	4.09 ± 0.71	3.54 ± 0.45	3.67 ± 0.52	3.66 ± 0.40	3.74 ± 0.58	0.001	0.62	0.92
Haematocrit %	33.9 ± 3.6	35.2 ± 4.8	31.2 ± 3.1	32.4 ± 4.2	32.2 ± 2.3	33.3 ± 4.1	0.022	0.45	1.00
WBC 10^3^/µl	6.56 ± 1.48	7.86 ± 3.27	4.99 ± 1.49	6.71 ± 3.82	6.06 ± 2.57	7.74 ± 3.09	0.34	0.11	0.97
Neutrophils 10^3^/µl	4.553 ± 1.020	5.400 ± 2.605	3.255 ± 0.756	4.123 ± 2.579	4.297 ± 2.600	5.502 ± 2.945	0.22	0.18	0.97
Neutrophils %	69.9 ± 7.8	67.2 ± 10.0	67.2 ± 14.3	60.2 ± 11.3	67.6 ± 14.8	67.9 ± 17.6	0.35	0.49	0.59
Lymphocytes 10^3^/µl	1.262 ± 0.635	1.557 ± 0.834	1.163 ± 0.681	1.575 ± 0.986	1.162 ± 0.654	1.369 ± 0.794	0.56	0.39	0.76
Lymphocytes %	18.6 ± 8.1	21.6 ± 9.1	22.0 ± 9.8	25.6 ± 12.1	21.6 ± 9.9	21.0 ± 14.8	0.53	0.60	0.80
Monocyte 10^3^/µl	0.5717 ± 0.1715	0.6773 ± 0.4346	0.4633 ± 0.3001	0.8532 ± 0.7178	0.4633 ± 0.3117	0.7568 ± 0.5660	0.95	0.13	0.66
Monocyte %	8.86 ± 2.34	8.53 ± 3.65	8.53 ± 4.75	11.60 ± 4.05	8.60 ± 5.55	9.43 ± 5.53	0.59	0.37	0.46
Eosinophil 10^3^/µl	0.00014 ± 0.00014	0.00022 ± 0.00025	0.00006 ± 0.00006	0.00014 ± 0.00012	0.00012 ± 0.00011	0.00012 ± 0.00018	0.27	0.38	0.62
Eosinophil %	2.03 ± 1.82	1.67 ± 2.30	1.17 ± 0.83	1.28 ± 2.92	1.74 ± 1.27	1.07 ± 1.89	0.62	0.67	0.84
Basophil 10^3^/µl	0.00004 ± 0.00001	0.00006 ± 0.00012	0.00005 ± 0.00004	0.00005 ± 0.00004	0.00004 ± 0.0002	0.0007 ± 0.00017	0.99	0.53	0.85
Basophil %	0.61 ± 0.14	0.58 ± 0.74	1.13 ± 0.97	0.67 ± 0.34	0.66 ± 0.67	0.77 ± 1.65	0.61	0.67	0.62
Platelets 10^3^/µl	233.000 ± 86.070	295.227 ± 117.576	299.500 ± 167.947	350.727 ± 226.317	210.833 ± 100.649	307.091 ± 177.309	0.37	0.18	0.90
Ratios									
NLR	5.06 ± 3.91	4.05 ± 2.74	7.73 ± 12.97	3.15 ± 1.90	9.32 ± 16.78	5.67 ± 4.34	0.08	0.21	0.37
MLR	0.68 ± 0.65	0.49 ± 0.32	0.44 ± 0.19	0.58 ± 0.32	0.38 ± 0.19	0.59 ± 0.40	0.64	0.63	0.14
PLTLR	258.2 ± 216.6	214.3 ± 102.0	640.3 ± 1035.8	251.5 ± 127.9	407.4 ± 647.8	252.0 ± 121.1	0.014	0.14	0.046

Statistical analysis: two-factor analysis of variance, the first factor (between factor) being group (mPDAC and laPDAC) and the second factor (within factor) being time (three measurements, TA, TB, TC), with repeated measurements in the time factor.

### Correlations among blood cell count subpopulation

#### Healthy subjects (controls)

In healthy subjects significant correlations were observed between levels of M and N (r = 0.53, p = 0.023), between WBC and N (r = 0. 91, p < 0.0001), M (r = 0.65, p = 0.004) and L (r = 0.54, p = 0.019), respectively. No significant associations were found between PLT and N, M, L, respectively. Lymphocytes correlated only with WBC (r = 0.54, p = 0.019).

#### PDAC patients

Table 7^66^ shows that at TA significant mutual correlations existed among N, P, M. At TB, the correlations of PLT with M and N, while being weaker than in TA, were still visible. M levels resulted significantly linked with L and N. At TC, PLT remained correlated with N and M. M correlated with L and to a lesser extent, with PLT. N correlated significantly with PLT.

**Table 7:** Relationships among peripheral blood cell counts in the study PDAC patients (n = 66). Only significant correlations with r ≥ 0.30 are reported^66^. The variables were correlated at the same time points (TA, TB, TC).

Monocytes			
TA	With		
	Neutrophils	r = 0.71	p < 0.0001
	Platelets	r = 0.54	p < 0.0001
TB	With		
	Platelets	r = 0.30	p = 0.014
	Lymphocytes	r = 0.54	p < 0.0001
	Neutrophils	r = 0.53	p < 0.0001
TC	With		
	Platelets	r = 0.33	p = 0.006
	Lymphocytes	r = 0.43	p = 0.00026

Neutrophils			
TA	With		
	Platelets	r = 0.57	p < 0.0001
	Monocytes	See above	
TB	With		
	Platelets	r = 0.33	p = 0.006
	Monocytes	See above	
TC	With		
	Platelets	r = 0.39	p = 0.001

Platelets			
TA	With		
	Monocytes	See above	p = 0.009
	Lymphocytes	r = 0.32	
	Neutrophils	See above	
TB	With		
	Monocytes	See above	
	Neutrophils	See above	
TC	With		
	Monocytes	See above	
	Neutrophils	See above	

Limphocytes			
TA	With		
	Platelets	See above	
TB	With		
	Monocytes	See above	
TC	With		
	Monocytes	See above	

Relationship between continuous variables assessed by Spearman’s rank correlation.

### Blood cell counts and mortality

Table 8 shows that patients who died < 6 months after diagnosis (< 6 mo), compared to patients who died > 6 months < 12 months (6–12 mo) after diagnosis, had higher baseline (TA) values of inflammatory indicators (NLR, MLR, PLTLR). At TB patients < 6 months showed increased N%.

**Table 8:** Blood immune cells variables (mean ± SD) in relation to patients times of death from diagnosis and survival.

Variables		Death < 6 mo n = 19	Death 6–12 mo n = 22	Survival > 12 mo n = 17	p value Anova	death < 6 mo *vs* death 6–12 mo p value	death < 6 mo *vs* survival > 12 mo p value	death 6–12 mo *vs* survival > 12 mo p value
Haemoglobin g/dl	TA	12.0 ± 1.9	12.0 ± 1.8	12.7 ± 1.2	0.53			
	TB	10.7 ± 1.3	11.4 ± 1.3	11.6 ± 1.5	0.14			
	TC	10.5 ± 1.5	11.8 ± 1.4	10.9 ± 1.4	0.032	0.010	0.40	0.10
WBC 10^3^/µl	TA	0.00824 ± 0.00291	0.00749 ± 0.00344	0.00681 ± 0.00190	0.34			
	TB	0.00761 ± 0.00295	0.00778 ± 0.00408	0.006 ± 0.00343	0.26			
	TC	0.00111 ± 0.0075	0.008.0 ± 0.0043	0.0065 ± 0.0023	0.035	0.063	0.013	0.40
Neutrophils 10^3^/µl	TA	6.243 ± 2.782	5.195 ± 2.521	4.581 ± 1.511	0.11			
	TB	5.498 ± 2.429	5.062 ± 3.161	3.850 ± 2.541	0.19			
	TC	9.057 ± 7.095	5.547 ± 4.067	4.665 ± 2.178	0.021	0.027	0.010	0.58
Neutrophils %	TA	72.6 ± 9.5	68.7 ± 9.3	66.3 ± 9.1	0.13			
	TB	72.8 ± 12.9	62.8 ± 12.1	62.0 ± 12.7	0.019	0.015	0.013	0.85
	TC	76.2 ± 15.5	63.9 ± 15.2	68.3 ± 11.7	0.028	0.008	0.11	0.34
Lymphocytes 10^3^/µl	TA	1.166 ± 0.599	1.460 ± 0.716	1.504 ± 0.496	0.20			
	TB	1.168 ± 0.681	1.612 ± 0.930	1.316 ± 0.590	0.17			
	TC	1.058 ± 0.502	1.631 ± 0.972	1.272 ± 0.486	0.042	0.013	0.38	0.13
Lymphocytes %	TA	16.0 ± 8.1	20.9 ± 8.2	22.8 ± 6.6	0.032	0.053	0.012	0.44
	TB	17.5 ± 11.1	23.1 ± 10.6	24.6 ± 10.2	0.11			
	TC	12.7 ± 9.2	24.2 ± 13.1	20.8 ± 9.7	0.005	0.002	0.031	0.35
Monocyte 10^3^/µl	TA	0.6874 ± 0.2341	0.6123 ± 0.3831	0.5424 ± 0.3495	0.43			
	TB	0.6294 ± 0.3489	0.8927 ± 0.5952	0.7076 ± 0.6937	0.32			
	TC	0.8779 ± 0.4152	0.9382 ± 0.8994	0.5629 ± 0.3534	0.17			
Monocyte %	TA	8.53 ± 1.87	7.78 ± 3.14	8.23 ± 4.35	0.76			
	TB	8.58 ± 3.28	11.27 ± 5.19	11.27 ± 5.53	0.15			
	TC	9.80 ± 6.11	9.42 ± 5.41	8.49 ± 4.63	0.76			
Eosinophil 10^3^/µl	TA	0.00011 ± 0.00010	0.00022 ± 0.00026	0.000015 ± 0.00012	0.13			
	TB	0.00062 ± 0.00243	0.00018 ± 0.00020	0.00010 ± 0.00008	0.47			
	TC	0.00007 ± 0.00007	0.00017 ± 0.00019	0.00012 ± 0.00011	0.12			
Eosinophil %	TA	1.08 ± 1.27	1.72 ± 2.21	1.47 ± 1.34	0.49			
	TB	1.14 ± 2.40	1.64 ± 2.98	1.18 ± 0.95	0.75			
	TC	0.74 ± 1.12	1.59 ± 2.11	1.53 ± 1.64	0.23			
Basophil 10^3^/µl	TA	0.00004 ± 0.00003	0.00006 ± 0.00012	0.00004 ± 0.00002	0.61			
	TB	0.00014 ± 0.00029	0.00005 ± 0.00004	0.00004 ± 0.00003	0.18			
	TC	0.00004 ± 0.00004	0.00007 ± 0.00017	0.00004 ± 0.00005	0.57			
Basophil %	TA	0.51 ± 0.47	0.54 ± 0.73	0.51 ± 0.27	0.98			
	TB	0.59 ± 0.44	0.67 ± 0.55	0.81 ± 0.53	0.44			
	TC	0.37 ± 0.35	0.81 ± 1.67	0.60 ± 0.50	0.44			
Platelets 10^3^/µl	TA	260.684 ± 84.271	243.273 ± 121.083	244.588 ± 106.196	0.85			
	TB	313.444 ± 171.252	356.864 ± 159.480	368.176 ± 233.785	0.66			
	TC	284.737 ± 123.874	267.455 ± 178.661	254.412 ± 123.595	0.82			
Ratios								
NLR	TA	7.23 ± 5.21	3.99 ± 2.18	3.26 ± 1.38	0.001	0.003	0.0008	0.50
	TB	7.22 ± 7.41	5.35 ± 11.20	3.32 ± 2.22	0.37			
	TC	10.7 ± 10.0	6.9 ± 14.5	4.4 ± 3.0	0.21			
MLR	TA	0.79 ± 0.53	0.42 ± 0.22	0.38 ± 0.23	0.001	0.002	0.001	0.73
	TB	0.61 ± 0.38	0.56 ± 0.32	0.52 ± 0.34	0.71			
	TC	0.88 ± 0.41	0.72 ± 0.86	0.46 ± 0.31	0.12			
PLTLR	TA	281.6 ± 158.4	183.4 ± 85.3	172.3 ± 77.8	0.007	0.007	0.005	0.76
	TB	459.1 ± 607.4	318.7 ± 425.7	295.2 ± 151.4	0.48			
	TC	346.7 ± 354.9	209.1 ± 150.1	232.2 ± 164.8	0.17			

Statistical analysis: ANOVA followed by post-hoc analysis (Tukey’s HSD test) in the case of significant differences.

At TC, patients who died < 6 months after diagnosis, had higher N, low L%. No differences were found between patients with survival 6–12 months and patients who survived > 12 months after diagnosis.

**Fig. 1:**
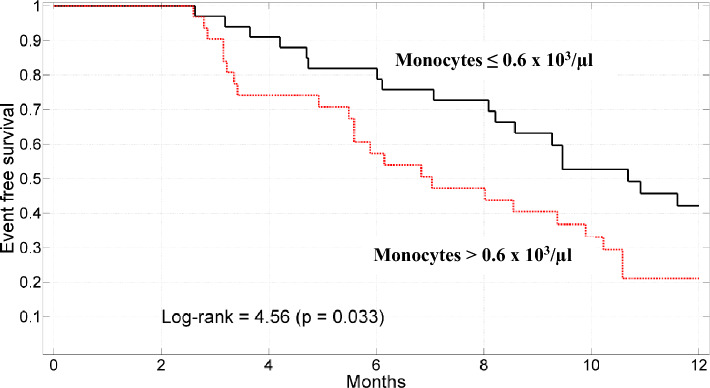
Kaplan–Meier survival curves stratified according to monocytes ≤ 0.6 × 10^3^/µl or monocytes > 0.6 × 10^3^/µl.

Figure 1 depicts Kaplan–Meier survival curves according to pretreatment (TA) monocytes count (cutoff value 0.6 × 10^3^/µl). The 12-month mortality rate varied from 82 to 60% (monocytes > 0.6 × 10^3^/µl and ≤ 0.6 × 10^3^/µl respectively, long rank = 4.56, p = 0.033).

**Fig. 2:**
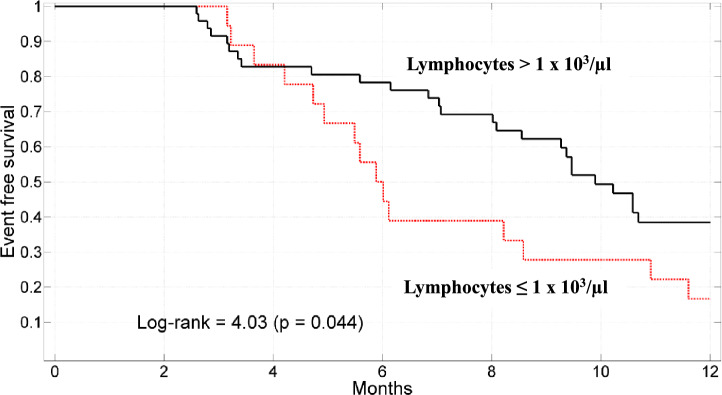
Kaplan–Meier survival curves stratified according to lymphocytes ≤ 1 × 10^3^/µl or lymphocytes > 1 × 10^3^/µl.

The mortality at 12 months for patients with pretreatment L ≤ 1 × 10^3^/µl was 83% vs 65% for L > 1 × 10^3^/µl (Fig. 2, Kaplan–Meier survival curves according to lymphocytes count, long rank = 4.03, p = 0.044).

**Fig. 3:**
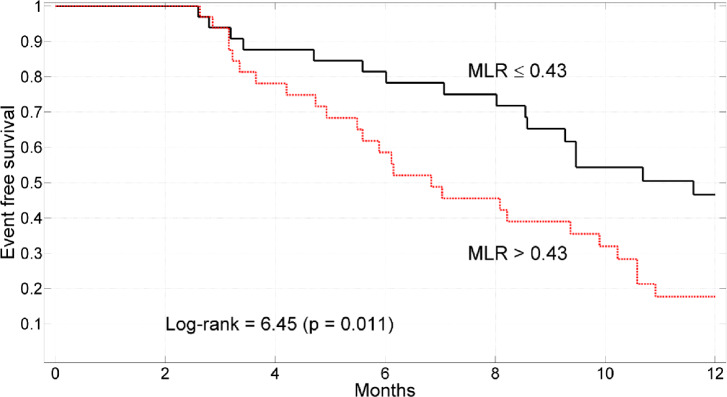
Kaplan–Meier survival curves stratified according to monocytes lymphocytes ratio (MLR) ≤ 0.43 or MLR > 0.43.

Finally, with pretreatment MLR > 0.43 the mortality was 83% vs 57% in case of MLR ≤ 0.43 (Fig. 3, log-rank = 6.45, p = 0.011).

### Anaemia at the diagnosis and during therapy

At the pretreatment period, the entire patient population presented a slight anaemia (Table2) that worsened during treatment phases (TB, TC).

## Discussion

The study demonstrates that three cycles of GnP treatment led to increased blood inflammatory cells, primarily reflected in elevated ratios of inflammatory cells to circulating lymphocytes. These inflammatory changes varied by tumour phenotype (mPDAC vs laPDAC) and gender. GnP treatment did not exacerbate pre-treatment lymphopenia but amplified the systemic inflammation and immune dysfunction already present at diagnosis. Anaemia progressively worsened during treatment, irrespective of cancer type or gender. Additionally, pre-treatment inflammatory biomarkers were higher in patients who survived less than six months compared to those who survived at least six months or more.

### Male group: baseline (TA) circulating immune cells and GnP-induced alterations in mPDAC *vs* laPDAC

At diagnosis, both mPDAC and laPDAC patients exhibited a systemic inflammatory state characterized by elevated NLR, MLR, and PLTLR, indicating a dominance of innate immune responses over adaptive immunity^67,68^. This suggests ongoing neutrophil and monocyte-driven cancer cell invasion, proliferation, metastasis^69,70^, and evasion of immune surveillance^71^.

The elevated MLR in particular points to a pro-tumorigenic activity facilitated by monocyte-neutrophil interactions that suppress the autoimmune response against tumour cells^70^. The findings support the hypothesis that GnP treatment significantly increased circulating monocyte counts in mPDAC patients, unlike in laPDAC, where monocyte levels remained stable or decreased. Elevated circulating monocyte counts have also been observed in other malignancies, including stage III colon cancer^27^. The temporal profiles of monocytes in mPDAC and laPDAC paralleled the trends seen in WBC counts for these two cancer phenotypes. This, combined with the absence of reductions in other white cell subpopulations, indicates that the increase in monocytes in mPDAC was genuine. It is likely that GnP treatment stimulated the tumour microenvironment (TME) to induce bone marrow to specifically enhance monocyte production. In cancer patients, circulating monocytes serve as indicators of tumour activity, as resident tumour-associated macrophages (TAM) correlate with blood macrophage percentages and reflect tumour burden^70^.

We hypothesize that the elevated M% observed in this study might result from a mismatch between the GnP-induced overload of killed cancer cells and an insufficient number of resident macrophages to fully clear the debris.

Gemcitabine, when combined with nab-Paclitaxel, exhibits more potent antitumour activity compared to gemcitabine alone, owing to its greater tumour penetration capacity^42,43^. This combination thus confers longer survival compared to gemcitabine alone^44,72^. Notably, monocytic myeloid-derived suppressor cells, the immature form of circulating monocytes, are not affected by gemcitabine^73^. This suggests that the increased M% was not due to a rebound effect during the treatment’s resting phase^73^.

PLT levels were another cell population that increased with GnP treatment, with a greater rise observed in mPDAC compared to laPDAC. The mutual influence between cancer and PLTs places PDAC patients, particularly those with mPDAC in this study, at higher risk for poor outcomes.

Cancer cells can activate PLTs and stimulate aggregation via thrombin and thromboxane A2 secretion^74,75^ and via ADP-stimulated P2Y1 and P2Y12 receptors^76,77^ and, indirectly, inducing PLT-granulocyte interactions^78^ and activating G proteins^78,79^. This helps explain the positive correlation between PLTs and neutrophils observed in this study. Activated PLTs, in turn, exert pro-tumorigenic effects, promoting the survival of metastatic cells in circulation^80^. Indeed, activated PLTs protect circulating cancer cells within thrombi^81^ from cytolysis by natural killer cells^82^ and from high shear stress^64^. Cancer-induced PLT aggregation has been demonstrated in several cancers, including pancreatic, colorectal, and kidney cancers^83–85^. Importantly, cancer-induced PLT aggregation correlates with higher metastatic potential^86^. Cancer-driven PLT activation and aggregation may also contribute to venous thrombosis at distant sites from the tumour^64,87^ and visceral thrombosis, a frequent complication in cancer associated with poorer prognosis and survival^88^. Activated PLTs contribute to cancer-associated inflammation by regulating immune cell migration toward tumours^80^. Additionally, PLTs serve as acute inflammation markers^89–94^ and play a critical role in tumour growth, progression, and metastasis, with PLT selectins and integrins being major mediators^81^. Aspirin has been shown to reduce metastasis risk, particularly in colorectal cancer^95^. This study found that in cancer patients—but not healthy subjects—PLT levels positively correlated with circulating immune cells (neutrophils, monocytes) at all time points (TA, TB, TC), underscoring the pivotal role of PLTs in inflammation and innate immunity^96^.

Chemokines, contained in PLT α-granules, regulate leukocyte migration and phagocytosis^96^, while PLT-derived cytokines affect monocyte and neutrophil chemotaxis^97^. These cytokines also promote monocyte differentiation into macrophages, induce cytokine release (e.g., IL-1α, IL-1β, IL-6, TNF-α), and support monocyte adhesion to endothelium^98^.

As lymphocyte counts remained unchanged during GnP treatment, PLT-associated inflammation was inadequately counteracted by the adaptive immune response.

### Circulating Lymphocytes in mPDAC and laPDAC

Although not statistically significant, the time-course changes in lymphocytes mPDAC and laPDAC warrant consideration.

GnP-induced inflammation in mPDAC seemed to involve greater inflammatory cell participation, whereas in laPDAC, lymphocytes more actively counteracted inflammation, leading to a gradual decrease over time. Lymphopenia in both mPDAC and laPDAC males reports an inadequate adaptive immune response in pre-treatment and during GnP therapy, suggesting the need for strategies to stimulate lymphocyte proliferation and activity.

### Female patients: immune response at baseline and post-GnP

At diagnosis, female patients, like males, exhibited systemic inflammation (high NLR, MLR, PLTLR), lymphopenia, and a predominant innate immune response to cancer over adaptive one. In mPDAC females, the GnP-induced immune response mirrored that of males, with increases in monocytes, MLR, and PLTLR, though these changes were statistically insignificant compared to baseline. This suggests a diminished monocytic inflammatory response to GnP. In laPDAC, the immune response to GnP was marked by elevated PLTLR, more pronounced than in mPDAC. As lymphocyte and PLT counts remained relatively stable, the increased PLTLR appears to result from decreased lymphocytes rather than increased PLTs. The mPDAC response, particularly in males, predominantly involved monocytic generation, while laPDAC showed increased PLT proliferation. However, lymphocyte proliferation remained inadequate relative to innate immune cell changes. Overall, female patients demonstrated persistent adaptive immune response inadequacy throughout GnP treatment. The partial intergender differences in immune response to GnP may stem from the influence of sex hormones on the immune system. Immune cells express receptors for oestrogens (OR), androgens (AR), and progesterone (P)^99–101 PLEASE INSERT 101 [99-101] IS CORRECT^. Elevated levels of oestrogens and progesterone promote neutrophil proliferation^102–104^. Given that study patients were in menopausal or perimenopausal stages, the gradual decline in oestrogen levels could be associated with reduced neutrophil proliferation compared to fertile age, potentially contributing to reduced cancer-related inflammation. OR signalling may limit monocyte recruitment to tissues^105^, while reduced testosterone may enhance monocyte and macrophage production of proinflammatory cytokines^106–108^. Sex-based differences in circulating immune cells in mPDAC patients may also result from oestrogen’s effects on lymphocytes (acquired immunity). Reduced oestrogen production enhances T and B lymphopoiesis, as oestrogen suppresses these lymphocyte phenotypes^109^ and decreases NF-κB activation and proinflammatory cytokine IL-1 production^110^. These intersex differences in immune regulation may help explain the attenuated inflammation observed in females with mPDAC and laPDAC undergoing GnP treatment. In contrast, GnP elicited a more pronounced thrombocyte proliferation response in females with laPDAC.

### Lymphopenia and GnP treatment

Lymphopenia at diagnosis, commonly observed in pancreatic cancer patients^111–113^, indicates reduced immune surveillance, leading to a loss of inhibition of cancer cell proliferation and migration. This is in synergy with elevated inflammatory cells to promote tumour proliferation and metastasis. GnP treatment did not significantly alter blood lymphocyte counts; however, small reductions observed in males and females with laPDAC and in males with mPDAC after the third GnP cycle may be clinically relevant by allowing enhanced innate immune activity. Several factors may contribute to lymphopenia, including the cancer itself, low availability of the essential amino acid tryptophan (TRP), increased TRP metabolism, malnutrition, and inadequate nutrient intake. Cancer-related monocytes can suppress T-cell formation^27^ while recruiting regulatory T cells (Tregs), which are crucial for tumour immune evasion^114^. The positive correlation between monocytes and lymphocytes observed during treatment, but not pre-treatment, may partially reflect an association between monocytes and Tregs. Monocytes inhibit CD4^+^, CD25^+^ and CD8^+^, CD25− T-cell proliferation and activation^58^. Limited lymphocyte proliferative capacity may also result from impaired mitochondrial energy generation due to the binding of Programmed Death-1 (PD-1) to its ligands PD-L1/2, which inhibits mitochondrial function and alters cristae structure^115^. Mitochondria are essential for T-cell metabolism^116^. Notably, increased PD-1 levels in blood T cells of septic shock patients correlate with decreased T-cell proliferation, and PD-L1 blockade in sepsis prevents lymphocyte apoptosis^117^.

Another cause of lymphopenia may be a shortage of the essential amino acid tryptophan (Trp) resulting from increased activity of the enzyme indoleamine 2,3-dioxygenase 1 (IDO-1), which is overexpressed in tumour cells^118^. leading to T cell proliferation arrest^118^.

Lymphopenia might also result from chronic lymphocyte exposure to cancer antigens, leading to T cell activation and metabolic exhaustion, independent of PD-1 signalling and Tregs^116^.

Other contributors to lymphopenia may include malnutrition^113,119^, altered T cell metabolism^120^, and body weight, which correlates with circulating lymphocyte levels expressed as a percentage of white blood cells^112^.

### Predictors of survival

The study revealed a 62.2% mortality rate within 12 months of diagnosis, with 28.9% occurring within the first 6 months. The survival rate beyond 12 months was 37.8%. We observed that higher levels of circulating inflammatory cells during the pre-treatment and treatment phases were associated with earlier mortality^69^. This underscores the role of an unbalanced immune response, where excessive inflammation over adaptive immunity contributes to reduced survival. Pre-treatment monocytes ≤ 0.6 × 10^3^/µl, lymphocytes > 1 × 10^3^/µl, and a monocyte-to-lymphocyte ratio (MLR) < 0.43 were identified as independent survival predictors. Previous research highlighted the association between elevated peripheral monocytes and prognosis^121^. This study adds that pre-treatment monocytes ≤ 0.6 × 10^3^/µl positively predict survival. High monocyte levels suppress host immunity, impairing the ability to attack tumour cells^58^. This suppression of effector T cells explains the link between monocytes, aggressive tumour growth, and reduced survival^27^. Lymphopenia exacerbates the tumorigenic effects of elevated monocytes. It signals a failure in immune surveillance^111^ and is a negative prognostic factor, especially in advanced cancers^122^.

In the present study we show that lymphocytes < 1 × 10^3^/µl were associated with increased mortality, while levels ≥ 1 × 10^3^/µl correlated with improved outcomes. Lymphopenia increases mortality risk due to treatment toxicity. In PDAC patients, lymphocytes ≥ 29.7% of total WBCs predicted chemotherapy tolerance^112^. In colorectal cancer, lymphocytes enhanced survival by inducing tumour cell apoptosis and inhibiting cancer proliferation and metastasis^123,124^. By inducing cytotoxic cell death and cytokine production lymphocytes inhibit both cancer proliferation and metastatic capacity^125,126^.

The MLR reflects the balance between pro-tumorigenic and anti-tumorigenic immune activities. An MLR < 0.43 at diagnosis was a survival predictor. In oesophageal squamous cell carcinoma patients undergoing esophagectomy, an LMR (lymphocyte-to-monocyte ratio) ≥ 3.83 better predicted survival^70^. This is the reciprocal of MLR ≤ 0.261. Meta-analyses have also confirmed the prognostic significance of LMR in colorectal cancer^58^. Beyond cancer, MLR serves as a marker of inflammation and prognosis in conditions such as acute ischemic stroke^127^ and mood disorders^128^.

### Normal range of values of circulating immune cells. patients’ anaemia

Patients with PDAC experience a state of inflammation and an imbalanced immune response to cancer. For the purposes of this study, we deemed the laboratory’s normal reference ranges as less reliable for two main reasons. First, the laboratory’s lower normal limit for lymphocytes in cancer patients, particularly those with PDAC, is often too low^112^. Second, the laboratory does not provide reference values for inter-cellular ratios. Therefore, we chose to use values observed in healthy controls, as these more accurately reflect the immune condition of the subjects in a “real-world” setting.

Both male and female patients in the pre-treatment phase presented with mild anaemia, which progressively worsened over time (TA, TB, TC). It is highly likely that the cancer-associated inflammation, present both before and during treatment, contributed to the anaemia^129 –131^ Anaemia is unlikely to be due to nutritional deficiencies, as the mean corpuscular volume (MCV) remained normal both at baseline and during chemotherapy.

### Study limitations

This study has several limitations that require further exploration. Firstly, the research was conducted at a single centre and involved a relatively small number of patients, although the study population was homogeneous with respect to the chemotherapeutic agent used. Monocyte and lymphocyte subsets were not available because they are not part of our routine bio humoral diagnostic procedures. Monocytes are categorized into three subtypes: classical inflammatory monocytes, protective monocytes, and intermediate monocytes^27^. Elevated proportions of intermediate monocytes have been observed in PDAC patients, and these cells are implicated in tumour progression^27^. Lymphocytes are crucial for anti-tumour immunity, and understanding their subpopulations is essential, both in the pre-treatment and treatment phases. For example, gemcitabine alone increases the effector T cell: Treg ratio without affecting the proliferative capacity of effector T cells^73^, whereas monocytes suppress the proliferation and activation of both CD4+ and CD8+ T cells^58^. Additionally, we did not have data on patients’ nutritional intake, which limited our ability to assess how their dietary habits might have influenced their overtime body weight and immune cell populations. Due to the relatively small sample size, stratification into multiple subgroups may lead to underpowered comparisons. Consequently, the predictive value of the identified cut-off points should be interpreted with caution. These findings are exploratory in nature and require validation in larger, independent cohorts.

## Conclusions

This study demonstrates that 3-cycle GnP treatment elicited different immune responses in PDAC patients, influenced by cancer phenotype and gender. Specifically, mPDAC, particularly in males, showed increases in monocytes and PLT, while laPDAC in females exhibited a PLTLR response induced by GnP.

### Translational relevance to clinical settings

This study offers key insights for clinical practice:

1. At the time of pancreatic cancer diagnosis, an MLR < 0.43, blood lymphocyte counts > 1 × 10^3^/µl, and/or monocyte counts < 0.6 × 10^3^/µl may predict a better prognosis.
2. Within the first 12 months post-diagnosis, metastatic PDAC patients are likely to have a poorer prognosis compared to those with locally advanced cancer.
3. During the first three months of chemotherapy, systemic inflammation may be exacerbated, as indicated by increased monocyte and platelet levels in metastatic cancer patients (especially males), or by increased platelet levels in locally advanced cancer patients (especially females). This heightened immune imbalance may increase the risk of infection, cancer progression and recurrence.
4. Both metastatic and locally advanced cancer patients, regardless of gender, could benefit from antiplatelet drugs. Numerous studies have shown that platelets, even within the normal range, can support tumour progression^132^, metastatic spread^64^, and poor prognosis in pancreatic cancer patients^133,134^.
5. Blood lymphocyte counts in patients with locally advanced cancer should be closely monitored over time.
6. Physicians may recognize that lymphopenia could result from lymphocyte exhaustion, which may negatively impact patient responses to immunotherapy, such as treatment with PD-1 blockade agents.

### Future research

Current immunotherapy approaches for PDAC have yielded disappointing results. Based on the findings of this study, we propose further research investigating the effects of immunotherapy targeting the reduction of monocyte activity and proliferation^4^, particularly in metastatic patients. Additionally, we believe that nutritional interventions aimed at achieving a more balanced inflammatory and adaptive immune response could enhance the effectiveness of immunotherapy in PDAC patients. Specifically, we hypothesize that nutrition interventions may reduce monocyte egress from the bone marrow and platelet activity. A Mediterranean diet, for example, has been shown to decrease inflammatory monocytes^135,136^ and platelet function^137^. Moreover, we suggest that efforts should be directed at improving lymphopenia, which is a key factor in mitigating both cancer-induced and treatment-related inflammation. Lymphocyte proliferation and activity may potentially be modulated by manipulating their metabolism through exogenous nutrients^116,118–120,138^. Improving lymphopenia could help reduce systemic inflammation, offering potential therapeutic benefits for PDAC patients. At present, we cannot determine whether the increases in PTL observed during GnP treatment are due to GnP-induced cancer cell death/damage, contributing to systemic inflammation, or disease progression. This important question warrants investigation in future, well-designed studies.

## Data Availability

The data that support the findings of this study are available from the corresponding author upon reasonable request.

## Abbreviations

AR: Androgens
Contr: Controls
CT: Computed tomography examination
G: Gemcitabine
GnP: Gemcitabine-nab-paclitaxel
IDO-1: Indoleamine 2,3-dioxygenase 1
L: Lymphocyte
laPDAC: Locally advanced PDAC
M: Monocyte
MCV: Mean corpuscular volume
MLR: Monocyte lymphocyte ratio
mPDAC: Metastatic PDAC
N: Neutrophil
NLR: Neutrophil lymphocyte ratio
NO: Nitric oxide
OR: Oestrogens
P: Progesterone
PD-1: Programmed death-1
PDAC: Pancreatic ductal adenocarcinoma
PE4: Prostaglandin E2 receptor 4
PLT: Platelet
PLTLR: Platelet lymphocyte ratio
ROS: Reactive oxygen species
sEH: Eicosanoid hydroxylase
TA: Time A
TAM: Tumour associated macrophages
TB: Time B
TC: Time C
TME: Tumour microenvironment
Tregs: Recruiting regulatory T
TRP: Tryptophan
WBC: White blood cell
